# Remediating Reduced Autobiographical Memory in Healthy Older Adults With Computerized Memory Specificity Training (c-MeST): An Observational Before-After Study

**DOI:** 10.2196/13333

**Published:** 2019-05-14

**Authors:** Kris Martens, Keisuke Takano, Tom J Barry, Jolien Goedleven, Louise Van den Meutter, Filip Raes

**Affiliations:** 1 Faculty of Psychology and Educational Sciences KU Leuven Leuven Belgium; 2 Department of Psychology Ludwig-Maximilians-University of Munich Munich Germany; 3 Department of Psychology The University of Hong Kong Hong Kong China; 4 Department of Psychology The Institute of Psychiatry King’s College London London United Kingdom

**Keywords:** memory specificity training, autobiographical memory, cognitive aging, online, depression, memory, telemedicine, rumination, cognitive

## Abstract

**Background:**

The ability to retrieve specific autobiographical memories decreases with cognitive aging. This decline is clinically relevant due to its association with impairments in problem solving, daily functioning, and depression. A therapist-delivered group training protocol, Memory Specificity Training (MeST), has been shown to enhance the retrieval of specific memories while ameliorating the impairments and negative outcomes associated with reduced specificity. The therapist-delivered nature of this intervention means it is relatively expensive to deliver and difficult for people with mobility impairments, such as older people, to receive.

**Objective:**

The objective of this study was to test if a novel, Web-based computerized version of a group training protocol called Memory Specificity Training, has the potential to increase autobiographical memory specificity and impact associated secondary psychological processes.

**Methods:**

A total of 21 participants (13 female; mean age 67.05, SD 6.55) who experienced a deficit in retrieving specific autobiographical memory were trained with c-MeST. We assessed memory specificity at preintervention and postintervention, as well as secondary processes such as depressive symptoms, rumination, and problem-solving skills.

**Results:**

Memory specificity increased significantly after participants completed c-MeST (*r*=.57). Session-to-session scores indicated that autobiographical memory specificity improved most from the online baseline assessment to the first Web-based session. Symptoms or secondary processes such as problem-solving skills did not change significantly.

**Conclusions:**

A Web-based automated individual version of MeST is a feasible, low-cost intervention for reduced memory specificity in healthy older adults. Future studies should clarify the preventive impact of c-MeST in other at-risk sample populations with longer follow-up times.

## Introduction

### Background

The world’s rapidly aging population [1] poses several challenges for societies regarding whether they can develop scalable interventions for maintaining quality of life and independence among an increasingly older population. One important cognitive factor associated with cognitive aging is a decrease in the ability to retrieve specific, personal memories [2]. This factor, referred to as reduced autobiographical memory specificity (rAMS) or overgeneral autobiographical memory [3], is associated with depression [2], impaired problem solving [4], and difficulty maintaining independence [5]. The link between these processes can be explained by the constructive episodic simulation hypothesis, which states that similar episodic processes are central to retrieval of past memories and to construction and simulation of hypothetical events [6]. Consequently, people who can retrieve more specific memories are better able to simulate possible events; they are also better able to formulate solutions to problems that might emerge in their future and plan for how to implement these solutions.

rAMS was first studied in depression (see [3] for a review) and trauma [7], and is now considered a trait marker for depression [8]. The first attempt to remediate rAMS [9] involved a 4-session group training program called Memory Specificity Training (MeST). This intervention improved memory specificity and associated cognitive processes (problem solving, rumination, and hopelessness) in depressed female inpatients. Subsequent investigations showed similar effects of MeST on rAMS in other patient groups [10-12]. In a cluster-randomized controlled platform pilot trial among people with depression, Werner-Seidler and colleagues [13] found that MeST was associated with improvements in memory specificity compared with a group receiving psychoeducation and supportive counselling.

The core component of MeST resembles the Autobiographical Memory Test (AMT) [14] used to assess rAMS. In the AMT, participants are presented with cue words and instructed to retrieve specific memories that these cue words remind them of. In MeST, participants receive similar instructions with the exception that they also receive feedback on the specificity of their responses and instructions for how they might be more specific and more detailed. Exercises are completed during the sessions and as homework assignments. In addition to exercises with cue words, in a second kind of specificity exercise participants are instructed each evening to write down one or two memories of that day (with no cue words given). After retrieving a specific memory, participants are encouraged to retrieve details of this specific moment.

rAMS is also an age-related phenomenon in *healthy* older adults [2], and aging is shown to contribute more than depressive symptoms to rAMS in people older than 50 years [15]. As the ability to retrieve specific memories is considered to be a protective factor for mental health [16], Leahy et al [17] examined whether improving memory specificity was possible among healthy adults over 70 years of age. In their study, they compared 3 groups: a MeST intervention group; a life review group, which also emphasized the recall of specific life events but placed them within the broader context of a person’s life narrative; and a control group, which was asked to complete a workbook of cognitively stimulating activities not directly related to autobiographical memory (ie, crossword and Sudoku puzzles). Each intervention took 4 weeks, with a posttraining assessment in the fifth week and a follow-up 3 months later. Leahy and colleagues reported significant improvements in autobiographical memory specificity in the MeST and life review groups at posttraining relative to the control group. However, this effect was not found at 3 months’ follow-up. Neither intervention had an effect on depression symptoms, functional independence, or executive functioning, but improvements in memory specificity were significantly related to improvements in social problem solving in both intervention groups.

Remediating rAMS has been found to be beneficial for older adults [17,18]. However, as societies age dramatically, making in-person training accessible to this growing and diverse population, who may not have contact with health care providers or who may have mobility or independence problems, would be challenging. Translating MeST into a computerized individualized platform could offer promise as a solution to these challenges [19]. A recently designed computerized algorithm for scoring the specificity of written autobiographical memories [20] offers new possibilities given that memory specificity training might now be delivered in the absence of a therapist and at home. This scoring algorithm, which has demonstrated good agreement with human-expert scorings [20], was incorporated into a Web-based platform for memory specificity training such that memories are coded and feedback can be given [21]. In a proof-of-concept study with participants with rAMS (operationalized as scoring <50% on the AMT), this Web-based computerized version of MeST (c-MeST) improved rAMS after 2 weeks of training (consisting of 7 sessions of 5 to 8 trials each) and the effect was maintained at 2-week follow-up, compared with a no-training control group.

### Objective

In this study, we aimed to examine a Web-based, individually delivered c-MeST that exclusively consisted of specificity trials. In this version of c-MeST, we standardized sessions, as each session contained the same amount of neutral, negative, and positive valence cue words, and cue words were equivalent in valence/pleasantness, activity/arousal, power/dominance [22], and concreteness [23] among the sessions. As a result, we could obtain session-by-session specificity scores and observe each participant’s progress. This standardization of sessions is in contrast to the study by Takano and colleagues [21], which followed the standard in-group version of MeST that increased the difficulty in exercises as the session progressed (eg, retrieving two memories in response to a single emotional cue). In addition, we aimed to assess depressive symptoms, rumination, and problem-solving skills online at preintervention and postintervention.

We aimed to test whether c-MeST would remediate rAMS among older adults in terms of change from preintervention to postintervention and the trajectory of change from session to session. We also aimed to test the extent to which c-MeST was associated with change in secondary outcomes and, in particular, a decrease in depressive symptoms and ruminative brooding, and an increase in problem-solving skills. Additionally, we aimed to test the feasibility of c-MeST for older adults in terms of whether, and to what extent, participants completed the intervention, and to gather reports of their experiences with c-MeST.

## Methods

### Participants

We recruited participants between October 2017 and April 2018 via (1) a network of university-related organizations for older alumni, (2) the website of a public advisory body for older adults, and (3) an online forum of a commercial website targeting older adults. We allowed people to participate regardless of location, but in practice all participants lived in Belgium. The study was described to them as the evaluation of a Web-based training program for a memory problem associated with cognitive aging and that is known to be a general vulnerability factor for associated processes such as impaired social problem solving and depression. The only inclusion criterion mentioned in the description of the study was a minimum age of 50 years. After completing the survey, participants were entered into a lottery to win a shopping coupon (€20). Participants showing rAMS were contacted and invited to participate in the preintervention measurement, until 20 participants completed c-MeST. An extra exclusion criterion at preintervention measurement was not having Dutch as their native language. The study received institutional ethical approval from the Social and Societal Ethics Committee of the KU Leuven (approval number G201709932).

### Measures

#### Autobiographical Memory Test

We measured autobiographical memory specificity before and after training using an online version of the AMT [14]. Participants were instructed to retrieve a specific memory for each of 10 cue words (5 positive, 5 negative; presented in Multimedia Appendix 1). The instructions stated that the memory needed to be specific—that is, the event recalled must have happened once and lasted less than a day but did not have to be an important event. One example of a correct answer and two examples of incorrect answers were provided. Because the assessment was online, in contrast with earlier studies using an in-person verbal version of the AMT (eg, [9]) no practice trials and no feedback during the test could be given and no time limit was applied. The AMT was scored by the online classifier and manually by the fourth author (JG). When scores contradicted each other (382/2010, 18.91% of the entries), the first author (KM) checked the answers and made the final decision. We used 2 sets of cues, and although we matched both sets for imageability, familiarity, and emotional extremity [8], we administered them in counterbalanced order across the 2 test moments to avoid an effect of the cue words. For this study, we operationalized rAMS as a score lower than 70%, which we considered as a deficit in memory specificity to be remediated via training. Published studies have some variability in the inclusion criterion, from no inclusion [9-11,17] to scoring lower than 50% [21] or lower than 70% [13].

#### Depressive Symptomatology

We used the Patient Health Questionnaire-9 (PHQ-9) [24] to measure depressive symptomatology. The PHQ-9 is a 9-item self-report measure of depressive symptoms, scoring the 9 *Diagnostic and Statistical Manual of Mental Disorders (Fifth Edition)* Major Depressive Episode criteria based on the frequency with which they have been experienced in the past 2 weeks, from 0 (“not at all”) to 3 (“nearly every day”). Scores can range from 0 to 27. PHQ-9 showed good internal consistency with a Cronbach alpha=.76 at the preintervention measurement.

#### Rumination

The Ruminative Response Scale-Brooding subscale (RRS-Brooding) [25,26] is a self-report questionnaire consisting of 5 items measuring brooding from the 22-item Ruminative Response Scale [27]. The items on the brooding factor are considered to measure the maladaptive coping of passively comparing one’s situation with some unachieved standard. For example, participants are asked to rate how frequently they tend to think “Why do I always react this way?” or “Why do I have problems other people do not have?” on a scale from 1 (“almost never”) to 4 (“always”). Scores range from 5 to 20. Cronbach alpha at preintervention was good (alpha=.81).

#### Problem Solving

We measured participants’ problem-solving skills with an online Dutch version of the Stress Anxiety Depression version of the Means-Ends Problem-Solving Procedure (SAD-MEPS) [28]. the original Stress Anxiety Depression version of the Means-Ends Problem-Solving Procedure (MEPS) [29] consists of a series of short stories or interpersonal problem situations faced by a hypothetical protagonist. Each story starts with the protagonist facing a specific problem, which is immediately followed by a successful ending. Participants are asked to provide the middle part of each story by typing in strategies or means for solving the particular problem. We used an adapted format [28], consisting of 2 versions of each 3 scenarios: 1 depression-related, 1 stress-related, and 1 anxiety-related story. We used 2 sets of stories and administered them in counterbalanced order to avoid an effect of the difficulty of the stories. Answers were scored manually by one of the authors (JG) on 2 dimensions. First, in line with the original manual [29], we scored stories for the number of relevant means (ie, discrete sequenced steps that enable the protagonist to get closer to the stated goal). The more relevant means a participant mentions, the better. Second, in line with Marx et al [30], we also scored stories for their effectiveness from 1 (“totally ineffective”) to 7 (“very effective”). Total scores result from a mean of the scores on the 3 stories.

### The c-MeST Intervention

The Web-based c-MeST consisted of 9 sessions of 11 specificity trials, which is similar in dose to the original in-person MeST (99 specificity trials vs 104 specificity exercises; [9]). The original in-person MeST [9] consisted of 1 session each week for 4 weeks, with homework assignments for every day in between sessions. For this study, we instructed participants to train on 1 session every other day, resulting in 17 days of training. The 11 trials of each session, 9 with cue words of different valences, can be categorized into 4 types: 3 positive, 3 negative, 3 neutral, and 2 memories of the day (1 about a memory of yesterday and 1 about today, without cue words). In this version of c-MeST, we standardized the sessions, as each session contained the same amount of each type of trial, and cue words were equivalent in valence/pleasantness, activity/arousal, power/dominance [22], and concreteness [23] among the sessions. Multimedia Appendix 1 lists the cue words. The 9 sets of cue words were presented in a fixed order, but the order of the cue words was randomized within each session.

Participants completed each session on a Web-based platform that contained instructions and tips about autobiographical specificity, similar to the instructions of the AMT but providing more examples. In each of the 11 specificity trials, participants were asked to retrieve a specific memory. The website used the computerized scoring algorithm for the AMT [20] to score entries and to automatically give feedback on whether the entry was specific. The scoring algorithm showed good performance against expert-rated scores in discriminating specific versus nonspecific memories (area under the receiver operating characteristic curve >.90; [31]). If the entry was scored as not specific, participants received feedback stating that their answer was not specific enough, were reminded that they needed to provide a specific memory that occurred on as specific day and that occurred only once, and were encouraged to reenter the memory or another memory with greater specificity. If, despite the feedback, participants could not generate a specific memory within three attempts, the next cue word was presented automatically. If participants succeeded in providing a specific memory, positive feedback was provided and participants were invited to provide more spatiotemporal and contextual details on the next page (ie, “Where did it happen? When did it happen? How long did it take? Who else was there? What can you see, hear, smell or taste? What kind of day was it?”). Participants were instructed to fill out these details only if they had not already provided them in their initial memory entry. Participants could skip a cue word if they wished to do so. There was no time limit per question.

### Measures of Training Experiences

After each session, participants were asked 3 closed and 2 open questions regarding (1) to what extent they found that the offered words were helpful or easy for retrieving a specific memory (0 = “not easy at all, words are very difficult to retrieve memories for” to 10 = “very easy, words are very easy to retrieve memories for”), (2) to what extent they experienced the feedback provided by the software as correct (0 = “not at all, a lot of mistakes” to 10 = “very correct, no mistakes”), (3) to what extent they experienced the session to be acceptable in length (1 = “way too short” to 5 = “way too long”), (4) how they experienced the training, and (5) whether they had any other remarks.

### Procedure

In the first online assessment, participants received an informed consent form including a question asking whether they wanted to provide contact details to be invited for a follow-up study, in case their results made them eligible. After completing an online AMT, participants who showed rAMS (operationalized as a score <70% on the AMT) were contacted by telephone and invited to participate in this study. We explained to participants that they were selected on their score on the online AMT. We asked participants whether they (1) recognized rAMS in their daily functioning and (2) were interested in participating in a study exploring the possibility of remediating this phenomenon. Because of concerns about feasibility and dropout, we offered participants 2 options: (1) if they wished to receive instructions for face-to-face c-MeST, we invited them to visit the first author (KM) for an in-person conversation (n=9); if not, then (2) instructions were given by telephone or email (n=12). All instructions were provided by the first author, a clinical psychologist, who could potentially refer participants to the appropriate care in case they were worried about cognitive problems. In either case, participants received an email with a link to a preintervention measurement of secondary measures (SAD-MEPS, RRS-Brooding, and PHQ-9), a second informed consent form, and a link to c-MeST. Participants were instructed to complete 1 session every other day, which would result in a training period of 17 days. Each Web-based session contained questions on feasibility. After participants completed c-MeST, another email was sent with an invitation to an online postintervention measurement of memory specificity (AMT) and secondary measures (SAD-MEPS, RRS-Brooding, and PHQ-9). When all data were gathered, participants were provided feedback about their scores and were invited to provide extra feedback on feasibility.

### Analysis of Data

We scored c-MeST sessions as the number of trials for which the patient’s first answer was classified as a specific autobiographical memory, in accordance with the logic of the AMT, resulting in a maximum of 11 points per session. We tested memory specificity and secondary outcomes (depressive symptoms, rumination, problem-solving skills) for deviation from the normal distribution using the Kolmogorov-Smirnov test. Results suggested that at both time points (preintervention and postintervention), there were significant deviations from normality (AMT postintervention, *P*=.02; RRS-Brooding preintervention, *P*=.04; SAD-MEPS means postintervention, *P*=.02). For memory specificity per type of trial, the assumption of normality was not satisfied either (all *P*<.001). Therefore, we used nonparametric statistics for all analyses.

To analyze the impact of c-MeST on memory specificity and secondary measures, we used a Wilcoxon signed rank test. We assessed relations between variables and change in variables with a Kendall tau rank correlation. To compare scores on different types of trials, we used a Kruskal-Wallis test with post hoc Mann-Whitney *U* tests. We used IBM SPSS Statistics for Windows version 25.0 (IBM Corporation) for all analyses. Multimedia Appendix 2 shows raw data of memory specificity and secondary measures.

## Results

### Sample Characteristics

**Figure 1:**
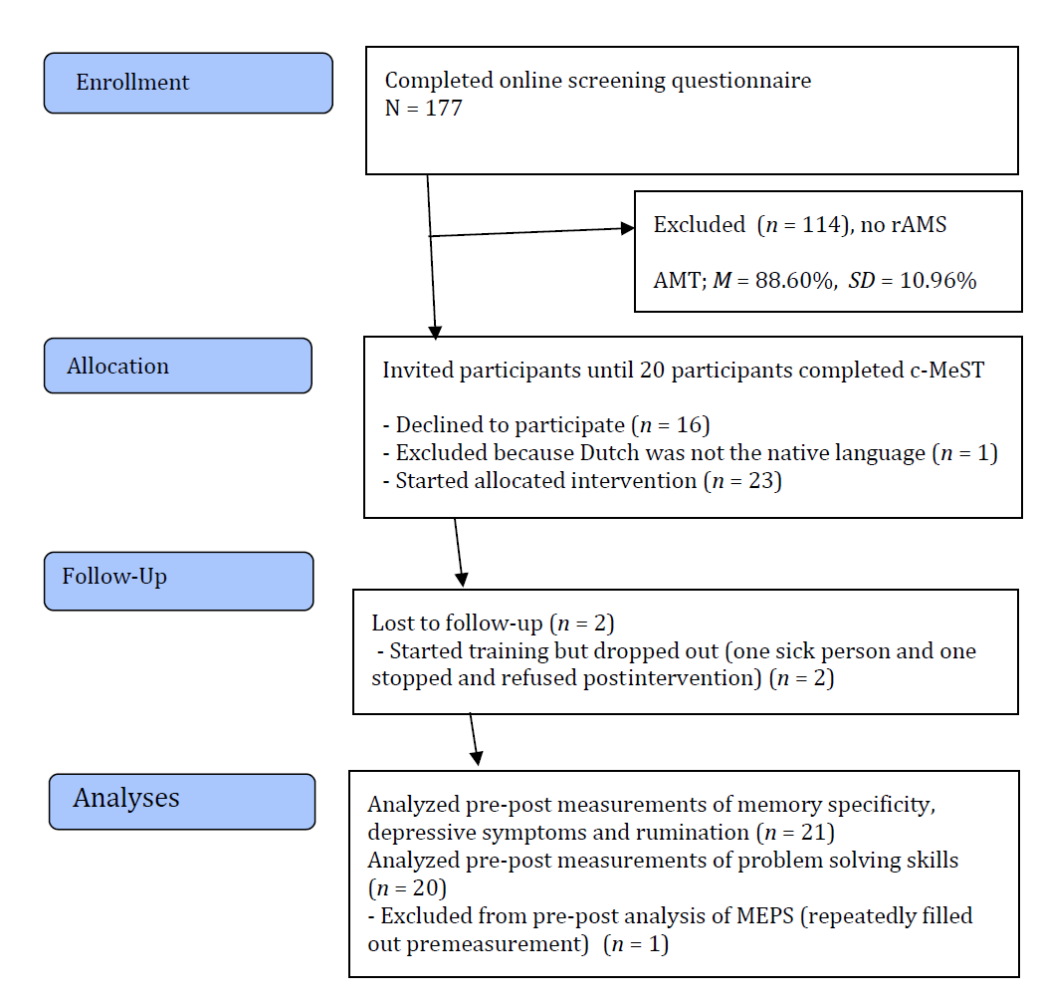
Flow diagram of the selection and inclusion process. AMT: Autobiographical Memory Test; c-MeST: computerized version of Memory Specificity Training; SAD-MEPS: Stress Anxiety Depression version of the Means-Ends Problem-Solving Procedure; rAMS: reduced autobiographical memory specificity.

In total, 177 participants aged 50 years and over (121 female; mean age 68.97, SD 6.60 years) filled out an online version of the AMT. This screening assessment identified 63 participants with rAMS, operationalized as a score of less than 70% on the AMT (mean 37.46%, SD 18.58%). Among them, we contacted 40 people to participate in this study. However, 16 people declined to participate and we excluded 1 person because Dutch was not their native language. The remaining 23 participants started c-MeST. During or after the training, 2 participants dropped out (1 person was sick and 1 person stopped during the training without a postintervention measurement). Finally, 21 participants (13 female) completed the postintervention measurements. Figure 1 shows a flow diagram of the selection and inclusion process.

Participants in c-MeST (n=21) were aged between 55 and 77 years (mean 67.05, SD 6.55 years). Participants’ age did not significantly correlate with memory specificity or any of the secondary measures (depressive symptoms, brooding, and problem solving) at preintervention measurement (with the biggest correlation being a Kendall tau correlation of –.25, *P*=.14 for brooding). At the preintervention measurement, 4 participants reported mild depressive symptoms (operationalized as a score of >5 on the PHQ-9) and 1 participant showed moderate depressive symptoms (score >10 on PHQ-9).

### Treatment Characteristics

A total of 2 participants did not complete c-MeST but did provide a postintervention measurement: 1 participant stopped after 4 sessions and 1 stopped after 5 sessions. We excluded 1 other participant from analyses of problem-solving skills, as they inadvertently filled out the preintervention assessment several times and thus also completed both versions of the SAD-MEPS task, which made a valid postintervention measurement impossible.

During c-MeST, participants needed to click the OK button after entering their memory, so that the memory was automatically scored, before filling out the details tab. Sometimes participants did not click OK and switched immediately to the details tab, which led to missing values in 1.67% of all memories scored. Participants were also allowed to skip a trial if they found it too difficult; they did so in 11.31% of the provided trials at a first attempt.

Participants were instructed to train every other day, but they were free to complete the sessions at another pace if they wished to. For the 19 participants who completed all sessions, the duration varied from 13 to 29 days (mean 18.37, SD 3.34 days). The number of days between the last session and the time of the postintervention measurement varied as well, between 0 and 16 (mean 3, SD 3.76 days).

### Check on Parallel Versions

There were no differences between the sets used for the AMT and SAD-MEPS, counterbalanced between participants across time points, and so subsequent analyses did not use counterbalance as a between-participants factor (see Multimedia Appendix 3).

### Memory Specificity

A Wilcoxon signed rank test showed that participants’ memory specificity increased significantly (*Z*=–3.70, *P*<.001) between preintervention (mean 30.00%) and postintervention (median 80.00%) as measured by the AMT, which can be regarded as a large effect size (*r*=.57). Comparing the group that received instructions on MeST in an in-person conversation (n=9) versus the group that received instructions by telephone or email (n=12), the groups did not differ significantly in terms of change in memory specificity between preintervention and postintervention measurements, assessed with a Mann-Whitney *U* test (*U*=47.00; *P*=.61).

**Figure 2:**
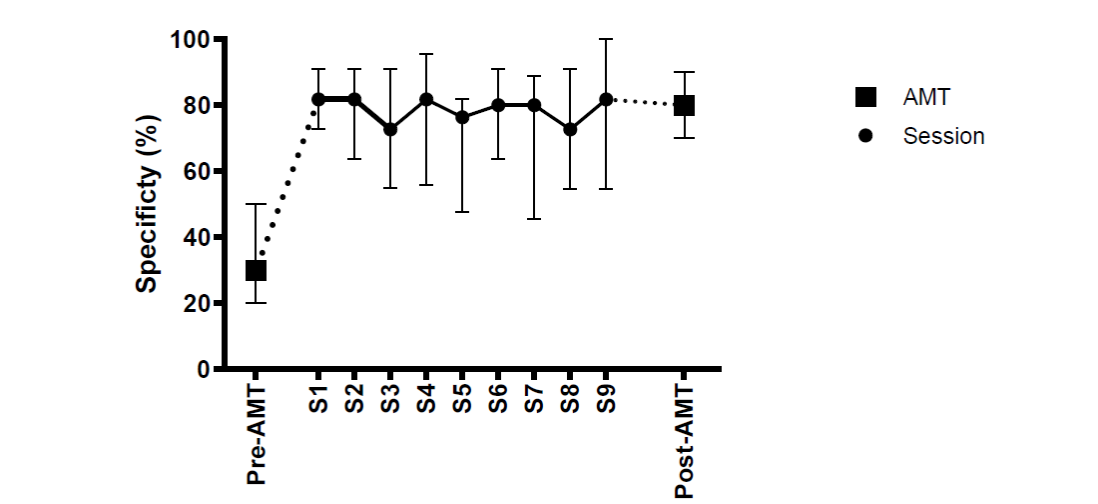
Median scores with interquartile ranges (25%-75%) of the Autobiographical Memory Test (AMT; pre- and postintervention measurements) and in between session-to-session scores on computerized version of Memory Specificity Training (c-MeST).

Session-to-session analyses, based on participants’ first attempts to retrieve a specific memory, showed that the proportion of specific answers given by participants improved significantly from the preintervention assessment (median 30.00%) to the end of the first session (median 81.82%; *Z*=3.95, *P*<.001, *r*=.61). We observed no further enhancement of specificity throughout the remaining sessions (varying from a median of 72.73% for sessions 3 and 8 to a median of 81.82% for sessions 1, 2, 4, and 9), as Figure 2 illustrates and Multimedia Appendix 4 shows.

As participants could give a correct answer in a second or even third attempt if they did not do so on a first attempt, we examined whether participants were successfully able to respond to the feedback given to them after their failed first attempt and to report a specific memory in their second or third attempt. Comparing the mean proportion of specific memories given on *first* attempts with the mean across *all* attempts, this increased from 73.24% to 78.36%. A Wilcoxon signed rank test revealed that this increase in memory specificity was statistically significant (*Z*=6.29, *P*<.001), which can be regarded as a large effect size (*r*=.54). Feedback helped participants to retrieve more specific memories.

**Table 1:** Median, range, and effect size using a Wilcoxon signed rank test for all variables at preintervention and postintervention assessments.

Variable	Preintervention		Postintervention		Effect (*r*)
	Median	Range (IQR^a^ 25%-75%)	Median	Range (IQR 25%-75%)	
AMT^b^	30.00	20.00-50.00	80.00	70.00-90.00	–.57
PHQ-9^c^	3.00	0.50-4.50	3.00	0.00-4.50	–.09
RRS-Brooding^d^	7.00	6.00-8.50	8.00	6.00-9.00	–.04
SAD-MEPS^e^-Means	2.00	1.42-2.92	1.67	1.67-2.92	–.07
SAD-MEPS-Effectiveness	4.50	3.33-5.33	4.83	3.67-5.33	–.03

^a^IQR: interquartile range.

^b^AMT: Autobiographical Memory Test.

^c^PHQ-9: Patient Health Questionnaire-9.

^d^RRS-Brooding: Ruminative Response Scale-Brooding subscale.

^e^SAD-MEPS: Stress Anxiety Depression version of the Means-Ends Problem-Solving Procedure.

To check whether certain trials were particularly hard to complete for participants, we compared scores (%) of participants for the 4 different trial types: trials with (1) neutral, (2) positive, and (3) negative cue words and (4) memories of the day. A Kruskal-Wallis test showed a significant difference in scores between different trials (χ^2^_3_=19.7, *P*<.001, with mean rank scores for neutral cues of 362.81; for positive cues, of 349.44; for negative cues, of 320.68; and for memories of the day, of 409.07). Post hoc Mann-Whitney *U* tests showed a statistically significant difference between scores on the category memories of the day (median 100%) in comparison with 3 other categories of exercises: neutral cues (median 66.67%; *U*=14038.50, *P*=.01), positive cues (median 66.67%; *U*=13509.50, *P*=.002), and negative cues (median 66.67%; *U*=12308.50, *P*<.001). Results indicated no significant differences in scores between types of cue words, but memories of the day can be regarded as the easiest type of trial.

In addition, analyses did not reveal that the number of days it took participants to fulfill the training (*τ*_b_=.10, *P*=.59) or the number of days between the last session and the postintervention measurement (*τ*_b_=.16, *P*=.38) significantly influenced the difference between preintervention and postintervention measurements of memory specificity.

### Changes in Secondary Outcomes

Participants reported low levels of depressive symptoms (median 3.00) and brooding (median 7.00) at preintervention measurement. As Table 1 shows, no significant change in reported depressive symptoms and brooding was evident by postintervention. In addition, we found no significant change in problem-solving skills (the number of means or the overall effectiveness of the solutions generated) between preintervention measurement and postintervention measurement (Table 1). Multimedia Appendix 3 shows exploratory analyses, in which we found no relevant association between change in memory specificity and change in secondary measures.

### Feasibility: Training Experiences

Overall, participants found the cue words used in each session to be of moderate difficulty (mean score 6.16, SD 2.18), and they experienced the classifier as correct more often than not (mean score 7.29, SD 1.89). The length of the sessions was experienced on average as “just right” and “a bit too long” (mean score 3.52, SD 0.80). Multimedia Appendix 5 shows mean scores on the 3 questions. For the open questions, 5 participants stated throughout the training that the rationale of the training was not clear, 4 participants experienced some technical problems, and 4 participants reported that they got better at retrieving memories more quickly.

## Discussion

### Principal Findings

This study examined the impact of Web-based memory specificity training (c-MeST) on difficulty retrieving specific autobiographical memories among healthy older adults. This proof-of-concept study showed that translating MeST to a Web-based application resulted in significant improvements in specificity.

Translating MeST to a Web-based application dismantled MeST to its core mechanism. In comparison with in-person, group MeST as used by Leahy and colleagues [17], in c-MeST the introductory session and therapist-plus-group interaction are absent. Other study protocols [13] included psychoeducation on memory problems in depression (session 1) and psychoeducation and exercises on how to notice when one is thinking on an overgeneral level in everyday contexts and how to tackle that (session 4). The results of this study support the idea that mere memory specificity trials are sufficient to improve AMS, which is in line with previous examinations of c-MeST in the context of depression ([21] and K Martens, MSc, TJ Barry, PhD, K Takano, PhD, P Onghena, PhD, F Raes, PhD, unpublished data, 2018). Session-by-session scores revealed an increase in specificity between the online preintervention measurement and the end of the first c-MeST session. A similar finding emerged in the only other MeST or c-MeST investigation to quantify change in specificity on a session-to-session basis (K Martens, MSc, TJ Barry, PhD, K Takano, PhD, P Onghena, PhD, F Raes, PhD, unpublished data, 2018). Critically, this previous investigation used a face-to-face assessment (using a version of the AMT that included feedback) in their preintervention assessment and then an online assessment at the end of their first session. The authors concluded that the rapid improvement in specificity may have been due to a change in modality between measurements. The fact that this sudden increase in memory specificity was observed again, but now with an online preintervention assessment of specificity (without feedback), refutes this suggestion. Instead, it seems that the effects of c-MeST on specificity are realized rapidly. In this study, the addition of automated feedback during the session in comparison with the premeasurement, might have contributed to the sudden increase in memory specificity. However, it remains unclear what dosage of c-MeST (how many sessions) is required for these effects to endure once the intervention ends.

Some discrepancies between specificity measured by the AMT and by c-MeST are also of note. The difference in cue words between AMT and c-MeST might explain why c-MeST evoked more specific memories. First, the addition of neutral cue words and memories of the day to assessments of specificity in c-MeST may have made it easier for participants to retrieve specific memories. Also, including participants with specificity scores lower than 70% at preintervention measurement may have caused the increase in scores at a second measurement to be due to regression to the mean [32]. Future investigations should test these possibilities by comparing c-MeST with a control intervention and by testing differences in specificity across different cue types within the AMT *and* c-MeST. Another interesting route for future investigations is to include a measure of speed (or response time) for each memory retrieval. A decrease in the response time to retrieve a specific memory over the training period may reflect an improvement in memory functions, which could better capture the training effect (or improvement trajectory) rather than the binary score of a specific memory.

Our hypothesis that c-MeST would lead to a decrease in depressive symptoms and rumination was not supported, but this may be due to floor effects for both variables. Participants’ scores at the preintervention measurement of depressive symptoms (PHQ-9 mean score 3.19, SD 2.96) fell in the range of scores found in the general population in this age range (from age 45 to >75: mean score 2.8, SD 3.5 to mean 4.4, SD 3.9; [33]). Scores on the rumination brooding scale were also in line with those found in the general population (mean score 7.62, SD 2.27 vs mean 8.6, SD 2.8; [34]). Leahy and colleagues [17] reported similar findings. It might, therefore, be unrealistic to predict further improvements from these low levels. It is of note that, among older adults who are vulnerable to subsequent increases in depression and impairments to quality of life and independence, the potential for c-MeST in preventing increases in these variables is worth further investigation.

We observed no increase in problem-solving skills. This might indicate that c-MeST does not influence problem-solving skills in healthy older adults with rAMS. This might also be explained by the use of an online version of SAD-MEPS, which is a test designed to be conducted face-to-face. After SAD-MEPS was used as a face-to-face measurement among people with depression [9], it was used as an online measurement among healthy students [35]. Both studies found no statistically significant effects from preintervention to postintervention in problem-solving skills. Future studies could assess problem-solving skills using measures that are more appropriate for online delivery or else the test should be conducted in person. The use of an adapted version of MEPS, SAD-MEPS, may not have been optimal for a group of healthy older adults with rAMS, and future research might use the standard MEPS.

The results of this study suggest that Web-based remediation of rAMS is feasible for older adults. Participants perceived the words to be moderately difficult, the feedback from the classifier as correct, and the length of the sessions as tolerable. However, participants varied in their preferences for session length and frequency. Given the nature of this research trial, we instructed participants to train in 9 sessions of 11 trials in 17 days. However, outside of a research context, participants should be able to train at their own pace. The software developed and tested here can enable participants to choose their own dosage and the frequency of training, which could further improve uptake and adherence. People also varied in scores for the 4 different kinds of trials. Future c-MeST could be personalized with an adaptive design, for example, by offering participants with low scores on one sort of cue words more of those similar trials. The software could also be combined with other instructions, such as those used in a life review, where specific memories are retrieved for particular life periods [18].

### Limitations

A limitation of this study is that we did not know the participants’ educational levels. We can assume that the average educational level was above average, as many participants were members of a university alumni group. Although internet use among older adults is generally high (in Belgium, 79% of older adults between 55 and 64 years of age have been reported to use the internet daily [36]), education and income levels are also positively correlated with internet skills [37]. Future investigations should examine the feasibility of c-MeST among a more diverse socioeconomic range of participants than we used. Another limitation is that we did not control for cognitive functioning. As previous research has indicated that specificity performance is associated with cognitive functioning such as executive functioning [38,39], future research should control for cognitive functioning. However, for this proof-of-concept study, the feasibility of c-MeST is promising.

### Conclusions

Web-based memory specificity training can effectively improve rAMS among healthy older adults. Translating the in-group training to a computerized version resulted in a feasible, scalable alternative, but we found no impact of this training on depressive symptoms, rumination, or problem-solving skills. Future investigations require follow-up assessments and control groups to assess the utility of c-MeST as an intervention for rAMS, and in the prevention of other negative outcomes such as increases in depression symptoms, among older adults.

## Appendix

Multimedia Appendix 1 Cue words used in assessments and training.

Multimedia Appendix 2 Raw data.

Multimedia Appendix 3 Exploratory analyses.

Multimedia Appendix 4 Specificity scores for each of the 9 sessions of computerized memory specificity training.

Multimedia Appendix 5 Results for 3 feasibility questions.

## Abbreviations

AMT: Autobiographical Memory Test
c-MeST: computerized version of Memory Specificity Training
MEPS: Means-Ends Problem-Solving Procedure
MeST: Memory Specificity Training
PHQ-9: Patient Health Questionnaire-9
rAMS: reduced autobiographical memory specificity
RRS-Brooding: Ruminative Response Scale-Brooding subscale
SAD-MEPS: Stress Anxiety Depression version of the Means-Ends Problem-Solving Procedure
