# Supplementary material for: Remediating Reduced Autobiographical Memory in Healthy Older Adults With Computerized Memory Specificity Training (c-MeST): An Observational Before-After Study
Source: J Med Internet Res. 2019 May 14;21(5):e13333. doi: 10.2196/13333 (PMC6538238; doi:10.2196/13333)
Supplement: Multimedia Appendix 1 [file jmir_v21i4e13333_app1.pdf]

Multimedia Appendix 1. Cue words used in assessments and training.

AMT A	AMT B	Session 1	Session 2	Session 3	Session 4	Session 5	Session 6	Session 7	Session 8	Set 9
Pleasant	Active	Pain	Accident	Captive	To lie	Ill	Rage	To stink	Quarrel	Odor
Mad	Furious	To cry	Angry	Dirty	Evil	False	Rot	Stupid	Sick	Ugly
Attentive	Interested	Broken	Weak	Wrong	Dirty	Lazy	Thick	Lost	Noise	Hunger
Hurt	Guilty	To work	Bank	Chair	Leg	Leaf	Table	Clock	Chin	Belly
Proud	Brave	Roof	Simple	Fish	Hot	Normal	Price	Letter	Hair	Tent
Angry	Helpless	Forest	Tree	Voice	Animal	News	Bicycle	Sauce	City	School
Social	Safe	In love	Holidays	Friend	Dear	Happy	Fun	Kiss	Merry	Feast
Clumsy	Sad	Handsome	Surprise	Free	Fun	Friendly	Smile	Funny	Laugh	Nice
Enthusiastic	Carefree	To kiss	Family	Smart	Well	Gift	Applause	Fine	Birthday	Music
Disillusioned	Anxious									

Note. AMT A, B= Autobiographical Memory Test, Set A and B (counterbalanced for the pre and post intervention assessments); Session 1 – 9 of the Computerized Memory Specificity Training.
