# Supplementary material for: Remediating Reduced Autobiographical Memory in Healthy Older Adults With Computerized Memory Specificity Training (c-MeST): An Observational Before-After Study
Source: J Med Internet Res. 2019 May 14;21(5):e13333. doi: 10.2196/13333 (PMC6538238; doi:10.2196/13333)
Supplement: Multimedia Appendix 3 [file jmir_v21i5e13333_app3.pdf]

Exploratory analyses

Check on parallel versions

Mann-Whitney tests indicated invariance between the two parallel AMT versions (n = 21) used as a pre-intervention measurement (p = .37) and as a post-intervention measurement (p = .59). Likewise, MEPS versions for both moments of assessment (n = 20) were not significantly different for the number of means (pre-intervention measurement: p = .50; post-intervention measurement: p = .66) or the overall effectiveness of generated solutions (pre-intervention measurement: p = .91; post-intervention measurement: p = .87). These results indicate that the different versions used for the AMT and MEPS, counterbalanced across the two test moments, can be regarded as equivalent.

Relation between change in memory specificity and change in secondary measures

Hypotheses: an increase in memory specificity from session-to-session was expected to correlate with a decrease in depressive symptoms and brooding, and an increase in problem solving skills.

The relations between the differences (by subtracting baseline from post-intervention) in pre- and post- intervention measurements of memory specificity, depressive symptoms, rumination and problem solving skills were examined. As Table A below shows, change in memory specificity did not correlate significantly with differences on other variables.

Also, we examined the relation between on the one hand session-to-session scores per participant of (1) memory specificity for *first* attempts, (2) memory specificity for *all* attempts, (3) mean number of attempts before retrieving a specific memory, (4) number of skipped trials, and on the other hand a change between pre- and post-measurements of

memory specificity and secondary outcomes. As shown in Table B, only the relationship between the trend of mean attempts needed to retrieve a specific memory over sessions and the increase in effectiveness of means of problem solving (SAD-MEPS) was statistically significant (τ_b = -.44, p = .018), which implies that needing less attempts throughout c-MeST is correlated with a bigger increase in effectiveness of problem solving.

Table A. Correlations (Kendall's tau) between differences of pre and post intervention scores of all variables

	Δ AMT	Δ PHQ-9	Δ RRS-5	Δ MEPS Means	Δ MEPS Effectiveness
Δ AMT	-	-.01	.12	-.21	.04
Δ PHQ-9		-	.14	-.18	-.01
Δ RRS-5			-	-.32	-.21
Δ MEPS					
Δ Means				-	.39*
Δ Effectiveness					-

Note. Δ = simple change score between pre and post intervention assessment; AMT = Autobiographical Memory Test; PHQ-9 = Patient Health Questionnaire 9; RRS-5 = The Ruminative Response Scale – Brooding subscale; MEPS (M) = Mean End Problem-Solving Task.

* $p < .05$ (2-tailed)

Table B. Correlations (Kendall's tau) between Session-To-Session Scores that Reflect In-Training Progress (Regression Coefficient) and Changes in Primary and Secondary Measures

	Δ AMT	Δ PHQ-9	Δ RRS-5	Δ MEPS Means	Δ MEPS Effectiveness
Specificity scores on *first attempts*	.29	-.04	-.04	.16	.01
Specificity on *all* attempts	.17	-.08	-.11	.19	-.06
Mean number of attempts before retrieving a specific memory	.09	-.15	.03	-.15	-.44*
Number of skipped exercises	-.06	.01	.31	-.28	-.03

Note. Δ = simple change score between pre and post intervention assessment; AMT = Autobiographical Memory Test; PHQ-9 = Patient Health Questionnaire 9; RRS-5 = The Ruminative Response Scale – Brooding subscale; MEPS (M) = Mean End Problem-Solving Task.

* $p < .05$ (2-tailed)
