# Supplementary material for: Remediating Reduced Autobiographical Memory in Healthy Older Adults With Computerized Memory Specificity Training (c-MeST): An Observational Before-After Study
Source: J Med Internet Res. 2019 May 14;21(5):e13333. doi: 10.2196/13333 (PMC6538238; doi:10.2196/13333)
Supplement: Multimedia Appendix 4 [file jmir_v21i5e13333_app4.pdf]

Multimedia Appendix 4. Specificity scores for each of the 9 sessions of computerized memory specificity training.

	Total score		
	n	*Md (%)*	*Range (Pc. 25 – 75)*
Session 1	21	81.82	72.73 – 81.82
Session 2	21	81.82	63.64 - 81.82
Session 3	21	72.73	55.06 – 72.73
Session 4	21	81.82	55.85 – 81.82
Session 5	20	76.39	47.73 – 76.39
Session 6	19	80.00	63.64 – 80.00
Session 7	19	80.00	45.45 – 80.00
Session 8	19	72.73	54.55 – 72.73
Session 9	19	81.82	54.55 – 81.82
