# Supplementary material for: Remediating Reduced Autobiographical Memory in Healthy Older Adults With Computerized Memory Specificity Training (c-MeST): An Observational Before-After Study
Source: J Med Internet Res. 2019 May 14;21(5):e13333. doi: 10.2196/13333 (PMC6538238; doi:10.2196/13333)
Supplement: Multimedia Appendix 5 [file jmir_v21i5e13333_app5.pdf]

Multimedia Appendix 5. Results for Three Feasibility Questions; (a) “have you found the offered words today easy to help you retrieve a specific memory?” (0 = not easy at all, very difficult words, 10 = very easy, easy words), (b) “have you experienced the feedback as correct?” (0 = not at all, a lot of mistakes, 10 = very correct, no mistakes), and (c) “have you experienced the session of today okay in length?” (1 = way too short, 2 = a bit too short, 3 = just right, 4 = a bit too long, 5 = way too long).

	Question 1 = difficulty words				Question 2 = correctness classifier				Question 3 = length of sessions			
	Min	***Max***	***M***	***SD***	***Min***	***Max***	***M***	***SD***	***Min***	***Max***	***M***	***SD***
Session1	2	9	6.47	2.37	1	10	7.32	2.08	3	6	3.53	0.77
Session 2	3	10	6.22	2.07	2	10	6.83	2.23	3	5	3.72	0.75
Session 3	1	10	5.74	2.38	3	10	7.42	1.61	3	5	3.53	0.77
Session 4	3	10	6.05	2.01	4	10	7.26	1.79	3	5	3.56	0.70
Session 5	1	10	6.06	2.29	3	10	7.11	1.84	3	5	3.33	0.69
Session 6	3	10	6.31	2.09	3	10	7.06	1.91	3	6	3.44	0.89
Session 7	1	10	6.13	2.26	5	10	7.67	1.76	3	6	3.67	0.98
Session 8	2	10	6.12	2.20	4	10	7.71	1.90	3	7	3.59	1.06
Session 9	1	10	6.35	2.34	3	10	7.29	2.08	3	5	3.31	0.60
**Total**			**6.16**	**2.18**			**7.29**	**1.89**			**3.52**	**.80**
